# Editorial: Complex network dynamics of cognitive processing in health and disease: current knowledge and future research

**DOI:** 10.3389/fncom.2026.1976480

**Published:** 2026-09-11

**Authors:** Simone Cauzzo, Camillo Porcaro, Chandan Kumar Behera, Suriya Prakash Muthukrishnan

**Affiliations:** 1 Parkinson and Movement Disorders Unit, Study Center for Neurodegeneration (CESNE), Department of Neuroscience, University of Padova, Padua, Italy; 2 Biomedical Engineering Research to Advance and Innovate Translational Neuroscience (BRAIN Unit), Department of Neuroscience and Padova Neuroscience Center, University of Padova, Padova, Italy; 3 Department of Humanities and Social Sciences, Indian Institute of Technology Ropar, Rupnagar, Punjab, India; 4 Department of Physiology, All India Institute of Medical Sciences, New Delhi, India

**Keywords:** brain connectivity, default mode network, edge-centric connectivity, functional connectivity network mapping, microstate dynamics, multimodal modeling

Over the past decade, neuroscience has undergone a profound conceptual shift: rather than ascribing cognitive functions to activations in isolated brain regions, the field increasingly recognizes that cognition emerges from the dynamic interplay of rapidly reconfiguring, distributed neural networks whose efficiency depends on flexible transitions, coordinated integration, and appropriate engagement of internally and externally oriented systems (Faskowitz et al., 2020; Novelli and Razi, 2022; Finc et al., 2026). Advances in neuroimaging and electrophysiology—including EEG, fMRI, MEG, PET, and multimodal combinations—have enabled researchers to characterize brain-network organization across complementary spatial and temporal scales, although each modality captures only part of the underlying dynamics (Lake and Higley, 2022). The evidence spans millisecond EEG states, moment-to-moment fMRI co-fluctuations, multimodal disease-network mapping, motor preparation, and neuroimmune/genomic interactions. The strongest convergence is that disease or increased cognitive demand changes network state transitions, not just regional activity. Simultaneously, advanced computational methods such as graph theory and machine learning offer new frameworks for understanding brain connectivity complexity (Mohammadi and Karwowski, 2024; Yang et al., 2025).

This Research Topic, “Complex Network Dynamics of Cognitive Processing in Health and Disease: Current Knowledge and Future Research,” was conceived to gather cutting-edge contributions examining how functional and causal interactions among brain regions underlie cognition in healthy individuals and how these interactions break down in neurological and psychiatric disorders. The five articles collected here span a broad clinical and methodological landscape—from Alzheimer's disease and Parkinson's disease to Post-Traumatic Stress Disorder (PTSD) and subjective cognitive decline (SCD)—and collectively illustrate both the power and the promise of network-level approaches to understanding the brain.

Two contributions in this Research Topic demonstrate that EEG microstates—brief, quasi-stable patterns of global scalp electrical activity lasting 80–120 ms—offer a uniquely sensitive window onto large-scale network dynamics (Michel et al., 2024).

Sayman et al. investigated resting-state microstate dynamics in Mild Cognitive Impairment (MCI), finding significantly increased Global Explained Variance for microstate classes B and C in MCI patients relative to healthy controls. Crucially, these elevations correlated negatively with scores on the Montreal Cognitive Assessment and Raven's Progressive Matrices, linking altered network dynamics directly to impairments in fluid reasoning and executive control. The authors propose that the overrepresentation of these microstates, recurring more frequently without becoming more stable, reflects reduced flexibility or maladaptive reorganization within the default mode network (DMN) and visual networks—an early signature of the network dysconnectivity that characterizes the predementia stage.

Yadav et al. extended microstate analysis into the motor domain, demonstrating that pre-movement EEG microstates encode graded information about the load to be lifted during a voluntary biceps curl task, indicating that network dynamics can encode the anticipated physical demands of an action before movement begins. Two specific microstate maps differentiated load conditions, with source maxima localized to the right insula and cingulate gyrus—regions implicated in interoceptive awareness, effort monitoring, and anticipatory motor control. These findings establish a compelling proof-of-concept for EEG microstate-based brain-computer interfaces capable of decoding motor intent before movement onset, with direct implications for assistive and neurorehabilitative technology.

Together, these two studies highlight a convergent message: microstate dynamics are not merely epiphenomenal resting-state fluctuations, but sensitive, millisecond-scale markers of both cognitive decline and motor preparation that conventional frequency-domain analyses would miss.

Complementary conclusions are reached in the remaining three articles leveraging functional and structural MRI to characterize network-level abnormalities in three distinct clinical populations.

Wei et al. applied an edge-centric functional connectivity framework to resting-state fMRI data from 211 patients with SCD and 210 healthy controls drawn from the Alzheimer's Disease Neuroimaging Initiative. By decomposing connectivity into moment-to-moment co-fluctuations—thereby bypassing the limitations of conventional sliding-window approaches—they identified reduced trough-to-trough duration (TTD) and lower peak amplitude in SCD, indicative of blunted and sluggish brain state transitions. Notably, TTD correlated positively with APOE ε4 gene dosage, suggesting a mechanistic link between amyloid/tau pathology and disrupted network flexibility even at the preclinical stage of Alzheimer's disease. The high-amplitude frame network proved particularly sensitive, identifying an SCD-related subnetwork of 11 brain regions and 13 edges—a potential biomarker constellation for early disease detection.

Yang et al. addressed neural network localization in Parkinson's disease (PD) with impulse control disorders (ICDs)—a clinically important but neurobiologically heterogeneous complication affecting up to 46% of PD patients. Using functional connectivity network mapping (FCNM) applied to a large normative connectome dataset (Human Connectome Project, *n* = 1,093), and integrating coordinates from 19 neuroimaging studies across multiple modalities, the authors identified convergent abnormalities in the DMN (middle and inferior temporal gyri, anterior cingulate cortex, angular gyrus) and the subcortical network (caudate nucleus). The network interpretation explains different regional findings: impulsivity results from disrupted coordination among self-referential processing, reward evaluation, cognitive control, and striatal action selection rather than from a single “impulsivity region.” It links abnormal DMN regulation and impaired task-related suppression to worse self-monitoring and decision-making.

Finally, Soliman et al. extend the network concept beyond neuroimaging. They propose that trauma-related memory processing and treatment response emerge from interactions among memory circuits, the hypothalamic–pituitary–adrenal axis, DMN-related cognition, immune signaling, epigenetic regulation, and B-cell function. Using artificial neural network (ANN) modeling and gene pathway analyses, they identified IL-10, IL-6, and Neuropeptide Y as key neuroimmune modulators linking B-cell function to PTSD remission, and proposed IL-10 as a potential early biomarker for PTSD onset. While methodologically distinct from the neuroimaging-centered contributions, this study enriches the Research Topic by introducing a systems-biology hypothesis: durable cognitive and emotional recovery may involve coordinated neural, endocrine, immune, and epigenetic reconfiguration rather than memory-network change alone.

Across these five contributions, several cross-cutting themes emerge. First, cognitive health depends on flexibility and on coordinated integration and segregation (Barabino et al., 2024). The DMN appears as a recurring locus of disruption—in MCI, SCD, PD with ICDs, and PTSD—underscoring its centrality to self-referential processing, memory, and cognitive control across the disease spectrum (Smallwood et al., 2021; Sanz-Morales and Melero, 2024). Interestingly, network dynamics emerge as predictive and context-sensitive features. Second, methodological innovation is driving discovery: edge-centric connectivity (Novelli and Razi, 2022), microstate analysis, and FCNM each offer complementary perspectives that conventional region-of-interest or seed-based approaches cannot provide. Third, the integration of genetics, immunology, and neuroimaging signals a necessary move toward multimodal and multiscale models of cognition and disease.

Looking ahead, longitudinal designs are critically required to determine whether the network signatures identified herein—including altered microstate dynamics, disrupted co-fluctuation patterns, and subcortical-cortical imbalances—precede clinical symptom onset and predict disease trajectory. Larger and more diverse cohorts will be essential to validate these biomarkers across different populations. The translation of EEG microstate and connectivity biomarkers into clinical tools—for early diagnosis, patient stratification, and treatment monitoring—represents a particularly exciting frontier (Michel et al., 2024). Finally, the integration of non-invasive brain stimulation approaches (TMS, tDCS) with real-time network-state decoding offers a pathway toward genuinely closed-loop, network-targeted interventions (Wischnewski et al., 2024; Casazza et al., 2026).
